# Comparison of the Chinese ischemic stroke subclassification and Trial of Org 10172 in acute stroke treatment systems in minor stroke

**DOI:** 10.1186/s12883-016-0688-y

**Published:** 2016-09-06

**Authors:** Sha Tan, Lei Zhang, Xiaoyu Chen, Yanqiang Wang, Yinyao Lin, Wei Cai, Yilong Shan, Wei Qiu, Xueqiang Hu, Zhengqi Lu

**Affiliations:** 1 Department of Neurology, The Third Affiliated Hospital of Sun Yat-sen University, No 600 Tianhe Road, Guangzhou City, Guangdong China; 2 Department of Neurology, The Fifth Affiliated Hospital of Sun Yat-sen University, No 52 Meihuadong Road, Zhuhai City, China; 3 Department of Neurology, People’s Hospital of Zhongshan City, No 2 Sun Yat-sen East Road, Zhongshan City, China; 4 Department of Neurology, Affiliated Hospital of Weifang Medical University, No 465 Yuhe Road, Weifang City, China

**Keywords:** Minor stroke, Diffusion weight imaging, Chinese Ischemic Stroke Subclassification, Trial of Org 10172 in Acute Stroke Treatment, Implications for treatment

## Abstract

**Background:**

The underlying causes of minor stroke are difficult to assess. Here, we evaluate the reliability of the Chinese Ischemic Stroke Subclassification (CISS) system in patients with minor stroke, and compare it to the Trial of Org 10172 in Acute Stroke Treatment (TOAST) system.

**Methods:**

A total of 320 patients with minor stroke were retrospectively registered and categorized into different subgroups of the CISS and TOAST by two neurologists. Inter- and intra-rater agreement with the two systems were assessed with kappa statistics.

**Results:**

The percentage of undetermined etiology (UE) cases in the CISS system was 77.3 % less than that in the TOAST system, which was statistically significant (*P* < 0.001). The percentage of large artery atherosclerosis (LAA) in the CISS system was 79.7 % more than that in the TOAST system, which was also statistically significant (*P* < 0.001). The kappa values for inter-examiner agreement were 0.898 (*P* = 0.031) and 0.732 (*P* = 0.022) for the CISS and TOAST systems, respectively. The intra-observer reliability indexes were moderate (0.569 for neurologist A, and 0.487 for neurologist B).

**Conclusions:**

The CISS and TOAST systems are both reliable in classifying patients with minor stroke. CISS classified more patients into known etiologic categories without sacrificing reliability.

## Background

Ischemic stroke is a major cause of worldwide neurological morbidity and mortality. Identifying the cause of ischemic stroke is of great value for therapeutic choice and prognostic evaluation [1–4]. The Trial of Org 10172 in Acute Stroke Treatment (TOAST) system is the most widely used classification system [5], but it is of limited utility in cases of “stroke of undetermined etiology (SUE)” and has modest inter-examiner reliability [6]. To overcome these issues, modifications to the original TOAST system have been made. For example, a Korean group proposed a new classification system for brain infarctions in 2007, which enriched the definitions of subtypes of ischemic stroke and proved to have higher reliability than the previous system [7].

The Chinese Ischemic Stroke Subclassification (CISS) system was proposed by Gao et al. in 2011 [8]. Compared with TOAST, CISS removed small artery occlusions (SAO), proposed penetrating artery disease, and further classified the underlying mechanisms of large artery atherosclerosis. These changes would be beneficial for the classification of minor stroke which is receiving increased attention in China. However, the reliability of the CISS categorization system has not been assessed. This study aimed to evaluate the reliability of the CISS in patients with minor stroke and to compare the performance of the CISS and TOAST systems in these patients.

## Methods

### Ethics statement

This research was approved by the ethics committee of the Third Affiliated Hospital of Sun Yat-sen University and conforms to the relevant regulatory standards. All participants involved in this study provided written informed consent.

### Patients

**Fig. 1:**
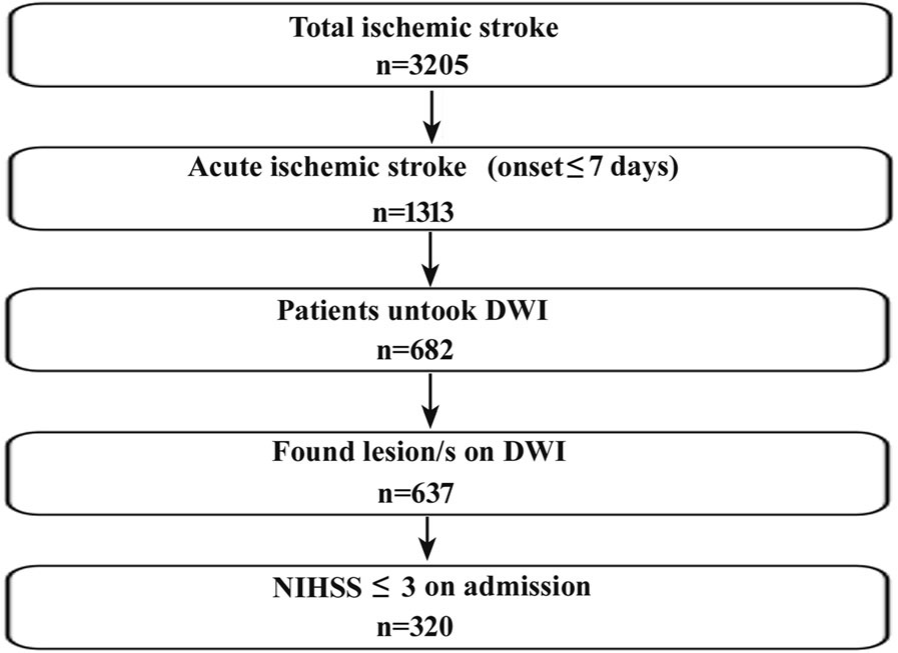
Patients inclusion chart. *DWI*: Diffusion weight imaging; *NIHSS*, National Institutes of Health Stroke Scale

The cohort was recruited retrospectively from the Department of Neurology of The Third Affiliated Hospital of Sun Yat-Sen University. In total, we screened 3205 consecutive patients with ischemic stroke admitted to our hospital between January 2008 and August 2013. Of these, 320 patients fulfilled the inclusion criteria: (a) onset age ≥18 years, (b) onset time ≤7 days, (c) lesions on diffusion weight imaging (DWI), and (d) National Institutes of Health Stroke Scale (NIHSS) score ≤3 on admission. The inclusion procedure is presented in Fig. 1. All patients underwent echocardiography (ECG) or 24 h ECG (94.4 %), high-resolution brain magnetic resonance imaging (HR-MRI), intracranial magnetic resonance angiography (MRA), DWI, and extra-cranial vascular imaging. Risk factors for stroke were also assessed, including gender, hypertension, diabetes, current cigarette smoking, coronary heart disease, previous transient ischemic attack (TIA) or stroke, and peripheral arterial disease.

### The subtype classification

Patients were classified into five etiologic/pathophysiological categories according to the TOAST system: large artery atherosclerosis (LAA), cardioembolism (CE), SAO, stroke of other determined etiologies (SOE), and SUE. To be diagnosed as LAA, patients should have paraclinical brain imaging findings of either significant (>50 %) stenosis or occlusion of large arteries, such as the internal carotid artery, middle cerebral artery, vertebral artery, and basilar artery, or their major branches, that is presumably caused by atherosclerosis. Diagnosis of CE requires paraclinical signs of a source of cardiac embolism. The category of SAO, which is often labeled as lacunar stroke, should have one of the traditional clinical lacunar syndromes and the HR-MRI examination can be normal or have a relevant lesion with a demonstrated diameter of less than 1.5 cm. In this category, the potential of LAA and CE should be eliminated. Patients with either more than one potential cause or no probable etiology were classified as stroke of undetermined etiology [5, 9].

**Fig. 2:**
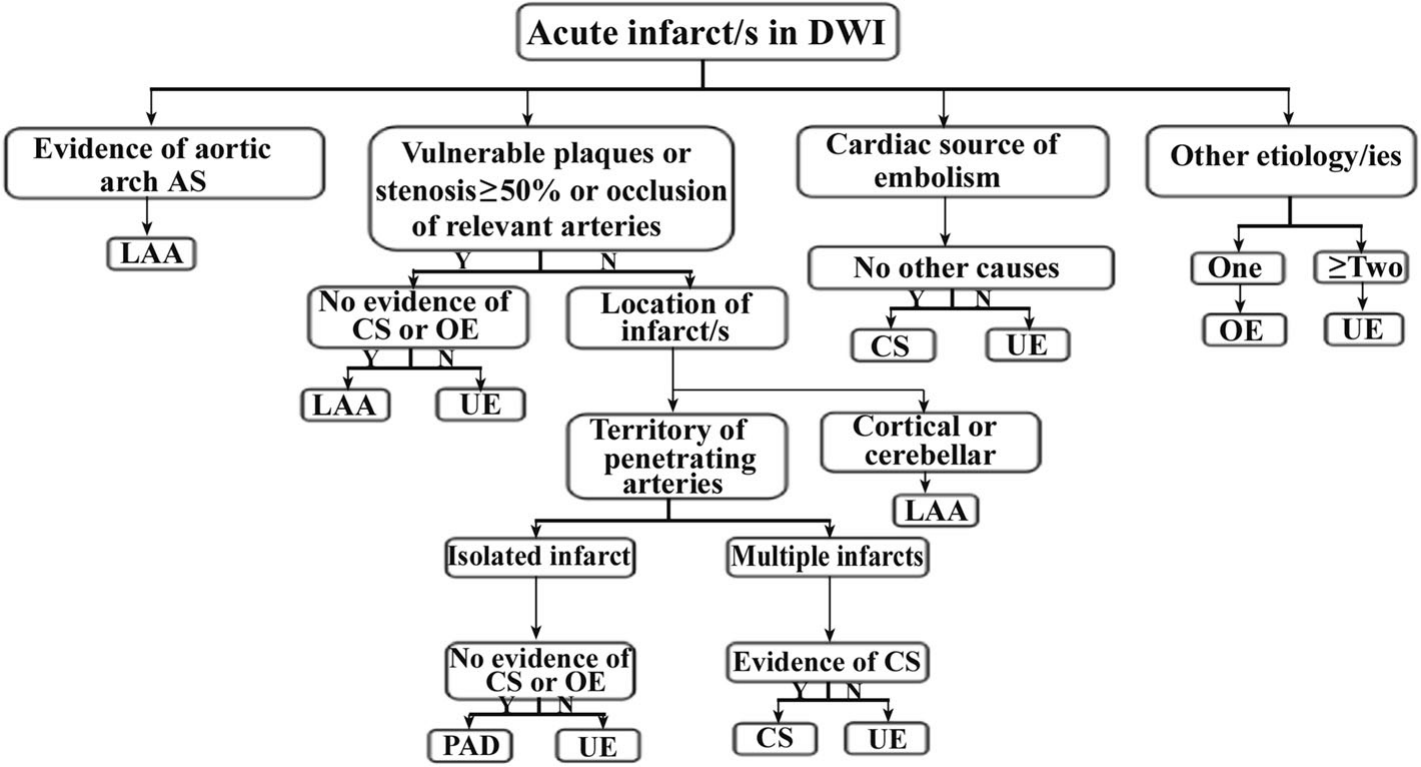
The flowgram of diagnosis of Chinese ischemic stroke subclassification. *LAA*, Large artery atherosclerosis; *CS*, Cardiogenic stroke; *PAD*, penetrating artery disease; *OE*, other etiologies; *UE*, undetermined etiology. Evidence of aortic arch atherosclerosis: aortic plaques >4 mm and/or aortic thrombi, detected by High-resolution magnetic resonance imaging/Magnetic Resonance Angiography (*HR-MRI/MRA*) and/or Transesophageal echocardiography (*TEE*)

CISS is a two-step system. The first step assigns patients into 5 etiology-based categories: LAA, cardiogenic stroke (CS), Penetrating artery disease (PAD), other etiologies (OE), and undetermined etiology (UE). The difference is that the CISS system further assigns intra- and extra-cranial LAA and PAD into categories on the basis of pathogenesis; something not done in the TOAST system. The second step is to further classify the underlying mechanism of ischemic stroke from the intracranial and extracranial LAA into the parent artery (plaque or thrombosis) occluding penetrating artery (PA), artery-to-artery (A-A) embolism, hypoperfusion/impaired emboli clearance, and multiple mechanisms [8]. Acute isolated infarction located in penetrating artery territory such as the basal ganglia or the pons is attributable to parent artery occluding PA, with evidence of plaque or stenosis in the parent artery. Multiple, scattered lesions in cortical and subcortical territories of relevant atherosclerotic vessel are usually in A-A subtypes. The diagnosis could be confirmed by transcranial doppler (TCD)–microembolic signals (MES) on TCD as to the single infarct. The hypoperfusion/impaired emboli clearance refers to the infarcts located in borderzone areas, usually accompanied with severely stenosed (>70 %) or occluded vessels. When there are two or more of the above mechanisms, we consider it to be multiple mechanisms. CISS also interpreted PAD as atherosclerosis at the proximal segment of the penetrating artery or lipohyalinotic degeneration of an arteriole. Two illustrations of CISS: First, a isolated penetrating artery infarct is classified in undetermined etiology if there is evidence of vulnerable plaques or stenosis ≥50 % in ipsilateral proximal intracranial or extracranial large arteries which includes carotid artery. Second, any other distribution of acute infarcts (except isolated infarct in the territory of one penetrating artery), with evidence of vulnerable plaques or stenosis ≥ 50 % in carotid artery or vertebral artery which supply the area of infarction would support the diagnosis of LAA. The operating procedure is presented in Fig. 2. Two neurologists classified all of the patients independently using TOAST and CISS criteria.

### Statistical analysis

Statistical analyses were performed with SPSS 19.0 (SPSS Inc., Chicago, IL, USA). Quantitative data were expressed as mean values ± standard deviations (SD) or median values (interquartile range), qualitative data were described by relative numbers. Independent proportions were compared using chi-square or Fisher’s exact tests as appropriate. Inter- and intra- agreement with the TOAST and CISS classifications systems were assessed with kappa statistics. k > 0.80 represented excellent agreement; 0.80 < k < 0.60 represented substantial agreement; 0.60 < k < 0.40 represented moderate agreement; 0.40 < k < 0 represented fair or poor agreement. The threshold for statistical significance was set at *p* < 0.05.

## Results

### Baseline characteristics of the patients with minor stroke

The clinical features of the patients with minor stroke are summarized in Table 1. The cohort comprised 203 men (63.4 %) and 117 women (36.6 %). The mean age was 64 years (range 28–91), the median NIHSS score on admission was 2 (range 0–3). Of these, 231 (72.2 %) had hypertension, 127 (39.7 %) had diabetes mellitus, 174 (54.4 %) had dyslipidemia, 29 (9.1 %) had coronary artery disease, 67 (20.9 %) had previous ischemic stroke (including TIA), 26 (8.1 %) had peripheral arterial disease, and 101 (31.5 %) were current smokers.

**Table 1:** Baseline characteristics of the patients of minor stroke

	TOTAL (*n* = 320)
Male gender, (n, %)	203, 63.4 %
Age,(y, mean ± SD)	64, ± 13
NIHSS score on admission,(median, IQR)	2,(1,3)
Hypertension, (n, %)	231, 72.2 %
Diabetes mellitus, (n, %)	127, 39.7 %
Dyslipidemia, (n, %)	174, 54.4 %
Coronary artery disease, (n, %)	29, 9.1 %
Previous stroke, (n, %)	67, 20.9 %
Current Smoker, (n, %)	101, 31.5 %
Peripheral arterial disease, (n, %)	26, 8.1 %

*SD* standard deviation, *IQR* interquartile range, *NIHSS* national institutes of health stroke scale

### Etiological subtypes of patients with minor stroke

As shown in Table 2, after classifying the patients with both the TOAST and CISS systems, we then compared the two. With CISS, 44.4 % (142/320) of the patients were classified into LAA, 4.1 % (13/320) into CS, 42.8 % (137/320) into PAD, 1.9 % (6/320) into OE, and 6.9 % (22/320) into UE. With TOAST, 24.7 % (79/320) of the patients were classified into LAA, 4.7 % (15/320) into CE, 38.4 % (123/320) into SAO, 1.9 % (6/320) into SOE, and 30.3 % (97/320) into SUE. The CISS system classified 77.3 % less patients into UE than the TOAST system, which was statistically significant (*P* < 0.001). Moreover, 79.7 % more patients were classified as LAA in the CISS system than in the TOAST system, which was also statistically significant.

**Table 2:** Comparison of TOAST and CISS subtypes, as performed by neurologist A

	TOAST		CISS		TOAST VS CISS	
	Frequency	Percent (%)	Frequency	Percent (%)	@	*P*
LAA	79	24.7	142	44.4	+79.7 %	<0.001*
CE/CS	15	4.7	13	4.1	−13 %	0.69
SAO/PAD	123	38.4	137	42.8	+11.3 %	0.26
SOE/OE	6	1.9	6	1.9	±	-
SUE/UE	97	30.3	22	6.9	−77.3 %	<0.001*
Total	320	100.0	*320*	100.0	±	-

*CISS* Chinese ischemic stroke subclassfication, *TOAST* trial of Org 10172 in acute stroke treatment, *LAA* large artery atherosclerosis, *CE* cardioembolism, *CS* cardiogenic stroke, *SAO* small-artery occlusion, *PAD* penetrating artery disease, *SOE* stroke of other etiologies, *OE* other etiologies, *SUE* stroke of undetermined etiology, *UE* undetermined etiology

@ indicate relative change between two classifications

*P < 0.001

### Reliability of the CISS and TOAST systems

Inter- and intra-reliabilities were measured by considering percent agreement of the two systems, and by using the unweighted *k* statistic. To evaluate the inter-rater reliability, two experienced neurologists independently assigned 320 patients to stroke subtypes on the basis of TOAST/CISS definitions. Intra-rater reliability was assessed by having the same observers classify the same 320 cases with the two different systems. The kappa value representing inter-rater agreement in the CISS system was 0.898 (*P* < 0.001, Tables 3, 7), and agreement between the two neurologists occurred in 266 of the 320 patients (83.1 %). While the reliability of the TOAST classification was 0.732 (*P* < 0.001, Tables 4 and 7), the two neurologists agreed on a subtype classification using the system in 261 of the 320 cases (81.6 %). Using both systems, observer A arrived at the same diagnosis for 158 of the 320 patients (49.4 %), and the observer B did this for only 134 (41.9 %). The intra-observer reliability indexes between TOAST and CISS criteria were 0.569 for neurologist A, and 0.487 for neurologist B (*P* < 0.001, Tables 5, 6 and 7).

**Table 3:** Distribution of CISS subtypes among the two neurologists

Neurologist B	Neurologist A										Total
	LAA-A	LAA-1	LAA-2	LAA-3	LAA-4	CS	PAD-1	PAD-2	OE	UE	
LAA-A	0	0	0	0	0	0	0	0	0	0	0
LAA-1	0	58	1	2	1	0	1	6	0	0	69
LAA-2	0	1	35	0	3	0	0	0	0	2	41
LAA-3	0	0	1	4	2	0	0	2	0	0	9
LAA-4	0	1	3	2	22	0	0	0	0	2	30
CS	0	0	0	0	0	13	0	0	0	0	13
PAD-1	0	1	0	0	0	0	19	11	0	0	31
PAD-2	0	2	0	0	0	0	6	92	0	1	101
OE	0	0	0	0	0	0	0	0	6	0	6
UE	0	0	2	0	1	0	0	0	0	17	20
Total	0	63	42	8	29	13	26	111	6	22	320

*CISS* Chinese ischemic stroke subclassfication, *LAA* large artery atherosclerosis,*LAA-A* aortic arch atherosclerosis, *LAA-1* parent artery (plaque or thrombus) occluding penetrating artery, *LAA-2* artery-to-artery embolism, *LAA-3* hypoperfusion/impaired emboli clearance, *LAA-4* hypoperfusion/impaired emboli clearance, *CS* cardiogenic stroke, *PAD* penetrating artery disease, *PAD-1* lipohyalinotic degeneration of arterioles, *PAD-2* atherosclerosis at the proximal segment of the penetrating arteries, *OE* other etiologies, *UE* undetermined etiology

*k* = 0.898

**Table 4:** Distribution of TOAST subtypes among the two neurologists

Neurologist B	Neurologist A					Total
	LAA	CE	SAO	SOE	SUE	
LAA	69	1	9	0	10	89
CE	2	10	2	0	1	15
SAO	5	1	105	0	16	127
SOE	0	0	0	6	0	7
SUE	3	3	6	0	70	82
Total	79	15	123	6	97	320

*TOAST* trial of Org 10172 in acute stroke treatment, *LAA* large artery atherosclerosis, *CE* cardioembolism, *SAO* small-artery occlusion, *SOE* stroke of other etiologies; SUE, stroke of undetermined etiology

*k* = 0.732

**Table 5:** Distribution of CISS and TOAST subtypes by neurologist A

CISS	TOAST					Total
	LAA	CE	SAO	SOE	SUE	
LAA	77	2	11	0	52	142
LAA-A	0	0	0	0	0	0
LAA-1	34	0	3	0	26	63
LAA-2	20	1	4	0	17	42
LAA-3	4	0	1	0	3	8
LAA-4	19	1	3	0	6	29
CS	0	13	0	0	0	13
PAD	1	0	109	0	27	137
OE	0	0	0	6	0	6
UE	1	0	3	0	18	22
Total	79	15	123	6	97	320

*CISS* Chinese ischemic stroke subclassfication, *TOAST* trial of Org 10172 in acute stroke treatment, *LAA* large artery atherosclerosis, *LAA-A* aortic arch atherosclerosis, *LAA-1* parent artery (plaque or thrombus) occluding penetrating artery, *LAA-2* artery-to-artery embolism, *LAA-3* hypoperfusion/impaired emboli clearance, *LAA-4* hypoperfusion/impaired emboli clearance, *CS* cardiogenic stroke, *PAD* penetrating artery disease, *OE* other etiologies, *UE* undetermined etiology, *CE* cardioembolism, *SAO* small-artery occlusion, *SOE* stroke of other etiologies, *SUE* stroke of undetermined etiology

*k* = 0.569

**Table 6:** Distribution of CISS and TOAST subtypes by neurologist B

CISS	TOAST					Total
	LAA	CE	SAO	SOE	SUE	
LAA	78	3	30	0	38	149
LAA-A	0	0	0	0	0	0
LAA-1	37	0	24	0	8	69
LAA-2	20	2	4	0	15	41
LAA-3	4	0	0	0	5	9
LAA-4	17	3	2	0	10	30
CS	0	10	1	0	2	13
PAD	9	1	96	1	25	132
OE	0	0	0	6	0	6
UE	2	1	0	0	17	20
Total	89	15	127	7	82	320

*CISS* Chinese ischemic stroke subclassfication, *TOAST* trial of Org 10172 in acute stroke treatment, *LAA* large artery atherosclerosis, *LAA-A* aortic arch atherosclerosis, *LAA-1* parent artery (plaque or thrombus) occluding penetrating artery, *LAA-2* artery-to-artery embolism, *LAA-3* hypoperfusion/impaired emboli clearance, *LAA-4* hypoperfusion/impaired emboli clearance, *CS* cardiogenic stroke, *PAD* penetrating artery disease, *OE* other etiologies, *UE* undetermined etiology, *CE* cardioembolism, *SAO* small-artery occlusion, *SOE* stroke of other etiologies, *SUE* stroke of undetermined etiology

*k* = 0.487

**Table 7:** Inter- and intra-agreement with CISS and TOAST systems

		Kappa	*P*
Inter-agreement	TOAST	0.732	<0.001*
	CISS	0.898	<0.001*
Intra-agreement	Neurologist A	0.569	<0.001*
	Neurologist B	0.487	<0.001*

Generally, *k* > 0.80 represents excellent agreement;0.80 < k < 0.60 is thought to be substantial agreement; 0.60 < k < 0.40,moderate agreement; 0.40 < k < 0.20, fair agreement; and *k* < 0.20, slight or poor agreement

*P < 0.001

## Discussion

Minor stroke is receiving an upsurge of attention in China. The CHANCE study showed that a combination of clopidogrel with aspirin is superior to aspirin alone for protecting against subsequent stroke in new TIA and minor stroke patients [10]. However, this study did not categorize the patients according to etiology, which is critical to individualized therapy. As combining the results of randomized controlled trials with individualized considerations may have therapeutic benefits, we believe that it would be beneficial for minor stroke patients to identify their underlying etiology.

The TOAST system is the most standard etiological classification system for ischemic stroke. As medical technology and clinical studies progress, the pathogenesis of ischemic stroke becomes more concrete. The CISS system represents a new etiological system for ischemic stroke [8]. A significant decline in subtypes of LAA and SVD in stroke/transient ischemic attack has been found in Western countries like England and Canada [11]. However, LAA, especially intracranial atherosclerosis (ICAS), is more common in Asian societies [12]. Furthermore, a high proportion of ICAS patients who experienced a minor stroke were at high risk of developing early neurological deterioration (END) [13]. The CISS system, which further classifies the underlying mechanism of LAA, might therefore be more appropriate for use with Chinese patients with minor stroke. However, the reliability of CISS has not been assessed. Here, we compared the CISS and TOAST systems in patients of minor stroke and examined reliability scores for them.

### CISS-etiologic category of minor stroke patients

In our study, 320 minor stroke patients were divided into five primary categories, consistent with the diverse causes and mechanisms known to underlie strokes. According to CISS, LAA was the largest subtype, which may be explained by the high prevalence of ICAS in China [14, 15]. However, CS was less than 5 % in our study, which is partly because most cardiac embolisms cause severe neurological deficits [16, 17] that results in an NIHSS score of above 3 on admission. Furthermore, there may have been limitations in our diagnostic tools. For example, transesophageal echo (TEE) and 30 events monitor or loop monitor are not routine procedures for ischemic stroke patients in our hospital.

### Distribution of etiology-CISS compared with TOAST

In our study, minor stroke patients were classified into diverse groups according to the etiology and mechanism of the disease. We found mismatches in LAA and UE with CISS and TOAST. In the TOAST system, most patients were classified into SAO, followed by UE and LAA, whereas in CISS, LAA and PAD groups took the largest proportion. This may reflect some differences in the definitions of subtypes across the two systems.

In our study, about half of the patients that were classified into SUE in TOAST were ascribed to LAA in CISS (52/97 in Neurologist A’s results, 38/82 in Neurologist B’s results). TOAST explicitly defines LAA with both specific stenosis degree of parent artery and size of lesion. As a result, if no other cause was found, the following two conditions would be classified as SUE using the TOAST classification: stenosis degree ≤ 50 % with the lesion diameter ≥ 1.5 cm; stenosis degree >50 % with the lesion diameter < 1.5 cm. By contrast, CISS does not include a restriction for stenosis degree or lesion diameter. Additionally, more than a quarter of the patients classified into SUE in TOAST were ascribed to PAD in CISS (27/97 in Neurologist A’s results, 25/82 in Neurologist B’s results). To help differentiate from lacunar stroke, CISS proposed the notion of PAD, as caused by atherosclerosis at the proximal segment of the penetrating artery or lipohyalinotic degeneration of an arteriole. The diagnosis of PAD was established for isolated penetrating artery territory infarct, when there was no evidence of atherosclerotic plaque or any degree of stenosis in the parent artery. The diagnostic procedure ignores the lesion size and clinical manifestation. Thus, compared with TOAST, CISS significantly decreased the number of patients classified as SUE, and might be more useful for clinicians to assess the etiologies and mechanisms of patients with minor stroke.

Differences between SAO and LAA were found to be the second discrepancy between TOAST and CISS, likely attributable to the different definitions of LAA used by the two systems. In TOAST, isolated PA territory infarct is classified into LAA only when both of the following conditions are included: i) brain imaging findings of either significant (>50 %) stenosis or occlusion of parent artery; and ii) the lesion is not smaller than 1.5 cm in diameter. By contrast, CISS further classifies LAA into four categories according to the underlying mechanism and emphasizes the significance of atherosclerotic plaque other than the lesion size. This means that proof of atherosclerotic plaque, any degree of stenosis in the parent artery, or new isolated PA territory infarcts should all be ascribed to LAA. Indeed, when other possible causes were excluded, patients with multiple small lesions (diameter <1.5 cm) in cortical or subcortical regions were attributed to SAO with the TOAST criteria, but classified into LAA in CISS if all the lesions were in the territory of the stenosed artery. In fact, the potential mechanism of this kind of infarct is artery-to-artery embolism [18], a branch of LAA with CISS. Therefore, a portion of SAO patients in TOAST were classified as LAA in CISS.

### Inter-rater reliability: CISS compared with TOAST

Both TOAST and CISS were found to be reliable in our study (inter-rater agreement: *k* = 0.732 for TOAST, *k* = 0.898 for CISS). CISS, which categorized the etiologic subtypes of minor stroke with excellent inter-examiner reliability based on assessment of clinical data obtained through medical record abstraction, showed higher practical utility. Although the TOAST classification has been widely used in prospective clinical trials and retrospective studies, its reliability was not always excellent or stable in previous studies [6, 19–21]. By contrast, the high inter-examiner agreement rates of the CISS suggest its potential utility in stroke research, though it should be noted that there are still some issues when applying CISS criteria. Differences in interpretation of medical records were a leading source of disagreement among examiners, and discrepant comprehension of subtype definition also contributed to the instability of CISS.

### Implications for treatment: CISS Classification

CISS is a more detailed etiologic system for ischemic stroke than TOAST, which means that it might be more practical for individualized treatment. As mentioned before, the underlying mechanism of patients with low-grade stenosis and small lesions is artery-to-artery embolism and/or impaired emboli clearance with CISS. For them, the root is the instability of plaques or thrombi, so intensive statin therapy may be beneficial. With regard to hypoperfusion cases, hypervolemic treatment is essential. However, we have to acknowledge that whether the impact of CISS on the schemes of therapy and secondary prevention could change clinical outcomes is still uncertain. Large prospective studies may be the only way to reach a definite conclusion.

### Limitations of study

There are some limitations in our research: (a) since we do not included the patients with moderate and severe strokes, our conclusions should not be generalized to the whole population of cerebral infarction; (b) as a retrospective study, bias is inevitable; (c) the insufficient diagnostic work-up could affect the accuracy of some results. For financial reasons, only a subset of patients took the examination of head computed tomography angiography (CTA), which is more precise in diagnosis of vascular stenosis. However, we do use high resolution MRA to increase the accuracy of the assessment. TCD emboli-monitoring and TEE are still not carried out in our hospital. The former is used to detect MES, especially in patients with a potential cardioembolic source, which is less common in minor stroke. Besides, a study with large cohort indicated that MES prevalence was relatively low in patients with a potential native cardioembolic source [22]. Therefore, the lack of TCD emboli-monitoring might not have a significant impact on our results. TEE could identify patent foramen ovale (PFO), although controversy still surrounds the issue of the relationship between PFO, paradoxical embolism, and cryptogenic stroke [23, 24]. Embolic Stroke is more likely to happen in PFO patients combined with venous thromboembolism [25, 26], which was not found in our objects. It therefore seemed reasonable to exclude the possibility that lack of TEE might significantly affect our results. Finally, we note there are some published studies that share the same insufficient diagnostic work-up with ours [20, 27]. In addition, a continuous heart monitor study could be required for definitive diagnosis of paroxysmal atrial fibrillation - a common cause of stroke, but for now it is difficult to implement because of the limited medical resource and economic cause.

## Conclusions

Our study shows several characteristics of the CISS and TOAST systems. Despite the lower reliability, TOAST has the advantage of simplicity, convenience and proficiency in practice. CISS enriches information about etiology and pathogenesis. In particular, CISS classifies more patients of minor stroke into known and precise etiologic categories, has higher reliability and may be more conducive to guide individual treatment. However, CISS requires more detailed examination, which will have financial implications. It is very important to note that this study was only conducted with minor stroke patients with NIHSS ≤3 and the results cannot be generalized to patients with moderate and severe strokes.

## Abbreviation

A-A: Artery-to-artery
CE: Cardioembolism
CISS: Chinese ischemic stroke subclassification
CS: Cardiogenic stroke
CTA: Computed tomography angiography
DWI: Diffusion weight imaging
ECG: Echocardiography
END: Early neurological deterioration
HR-MRI: High-resolution brain magnetic resonance imaging
ICAS: Intracranial atherosclerosis
LAA: Large artery atherosclerosis
MES: Microembolic signals
MRA: Magnetic resonance angiography
NIHSS: National institutes of health stroke scale
OE: Other etiologies
PA: Penetrating artery
PAD: Penetrating artery disease
PFO: Patent foramen ovale
SAO: Small artery occlusion
SOE: Stroke of other determined etiologies
SUE: Stroke of undetermined etiology
TCD: Transcranial doppler
TEE: Transesophageal echocardiography
TIA: Transient ischemic attack
TOAST: Trial of Org 10172 in Acute Stroke Treatment
UE: Undetermined etiology
